# Development, Validity, and Reliability of a Food Frequency Questionnaire for Omani Adults

**DOI:** 10.3390/nu17132220

**Published:** 2025-07-04

**Authors:** Tasnim Al Uraimi, Lyutha K. Al Subhi, Mostafa Waly, Mohammed Al Rizeiqi, Ruqaiya Al Balushi, Aaisha Al Kharusi

**Affiliations:** Department of Food Science and Nutrition, College of Agricultural and Marine Sciences, Sultan Qaboos University, P.O. Box 34, Al-Khodh 123, Oman; taluraim@gmail.com (T.A.U.); mostafa@squ.edu.om (M.W.); ruzeiki@squ.edu.om (M.A.R.); r.albalushi@squ.edu.om (R.A.B.); a.alkharusi3@squ.edu.om (A.A.K.)

**Keywords:** Omani, Arabic, cultural, food frequency questionnaire, reliability, validity, dietary assessment

## Abstract

**Background/Objectives:** Currently, Oman lacks a valid culture-specific food frequency questionnaire (FFQ) for adults. This research aimed to develop and validate a reliable (FFQ) specifically for Omani adults. The study was conducted in two phases, employing both qualitative and quantitative methods. Phase 1 was the development of the (FFQ); in this phase the Diet History Questionnaire II (DHQ II) was adapted to develop the Omani Food Frequency Questionnaire (OFFQ), translated to the Arabic language, back-translated to English, pilot tested, and then refined to be used in the reliability study. Phase 2 was the reliability study, in this phase, the OFFQ was administered twice in Arabic with the second being filled one to two weeks after the first questionnaire. **Methods**: A convenience sample of healthy Omani adults (*n* = 62) was recruited from Sultan Qaboos University (SQU) employees and students. To assess the reliability of the OFFQ both internal consistency and test–retest reliability were assessed. Internal consistency was assessed by conducting Cronbach’s α test, while test–retest reliability was assessed by comparing the median intake of food groups by calculating weighted kappa (K_W_) and intraclass correlation coefficients (ICCs). **Results**: Weighted kappa (K_W_) between the two OFFQ administrations showed fair to moderate agreement with (K_W_) values ranging from 0.38 to 0.60 for questions assessing the frequency of intake. While the median (K_W_) values between the two OFFQ administrations ranged from 0.26 to 0.58 for questions assessing portion size. The majority of food groups showed moderate to good test–retest reliability with median (ICCs) for food groups ranging from 0.57 to 0.80 for frequency questions and from 0.40 to 0.82 for portion questions. **Conclusions**: In conclusion, the newly developed OFFQ was deemed valid for the Omani dietary culture and is a reliable tool that can measure habitual dietary intake among Omani adults as evidenced by the reliability coefficients ranging from moderate to good agreement for the most food items and food groups. Future studies are warranted to assess the relative validity of the OFFQ and the inclusion of diverse demographic groups and a larger sample size.

## 1. Introduction

The concept of diet quality is usually misinterpreted and difficult to accurately define. According to previous literature diet quality refers to the level of conformity of an individual’s diet with specific dietary recommendations [1]. Relatively monitoring food patterns is used to measure diet quality by evaluating their alignment with established dietary recommendations in addition to the variety of essential food components for healthy diets [2]. Nutritional epidemiological studies have assessed the correlation between diet quality and disease by analyzing the intake of individual nutrients or food items [3]. Although this approach can provide valuable information, it has some inherent limitations [3,4]. For instance, foods and nutrients are not consumed in isolation, but rather in meals combining a variety of foods and nutrients. Thus, identifying associations for individual items can be challenging because of the synergistic or antagonistic interactions between foods and nutrients [5,6]. As a result, holistic approaches that evaluate overall dietary patterns, rather than individual nutrients, have gained popularity [7].

Dietary patterns can be assessed using three different methods: (1) empirical (data-driven—a posteriori), (2) theoretical (content or hypothesis-driven—a priori), (3) hybrid (combination) methods [6,8,9,10]. Empirical methods are exploratory or population specific [11]. Moreover, they assess the actual diet of the population by collecting dietary information using different dietary assessment methods, including 24-Hour Dietary Recall (24-HDR), Food Frequency Questionnaire (FFQ), Food Record (FR), etc. Data-driven methods use different statistical approaches to derive dietary patterns, such as principal components analysis (PCA) and factor analysis (FA) [12].

Theoretical methods construct pattern variables based on available scientific evidence to create disease-specific dietary patterns that can be reproduced among different populations [11]. This approach relies on dietary indexes or scores, such as the Diet Quality Index (DQI), Healthy Eating Index (HEI), or Mediterranean Diet Score (MDS) to assess conformity with dietary guidelines or recommendations [12]. However, these scores or indexes overlook the nuanced interactions between multiple dietary factors [13]. Empirical and theoretical approaches explore different aspects of dietary intake. Applying them together may yield complementary results [12]. This can be achieved by utilizing hybrid methods, which are the most recent advances in diet pattern analysis and are both a priori and exploratory statistical approaches [13].

FFQs capture usual dietary intake over time and are scalable and practical for large-scale epidemiological studies, such as the Nurses’ Health Study [14,15], to guide policies and programs. Other dietary assessment methods are less likely to be used in such studies for their limitations. Diet histories are time consuming and require well-trained interviewers [16]. Additionally, 24-HDRs are time consuming, not cost effective, require several recalls, show over and under estimation of portion size, capture current and not usual intake [17].

### 1.1. Prevalence of Overweight and Obesity in Oman

Overweight and obesity are primarily caused by an imbalance between energy intake and energy expenditure [18]. Since 1970, Oman has experienced a rapid socio-economic development which resulted in increased food availability and decreased physical activity [19,20]. According to a STEPwise NCD risk factor survey (STEPS) conducted in Oman, the prevalence of physical inactivity is 39%, low consumption of fruits and vegetables is 61%, consumption of alcohol and tobacco are 9% and 2%, respectively [21]. Arab speaking countries, including Oman, have adopted a more Westernized diet high in fat, added sugar, and carbohydrates [22]. These changes in lifestyle behaviors have affected Omani dietary patterns, resulting in increased rates of obesity. The prevalence of overweight and obesity was assessed in 17 Middle Eastern countries, and Oman had the highest prevalence of obesity (67.8%) between the years 2014 and 2020 [23].

### 1.2. Non-Communicable Diseases in Oman

A significant shift in the health status of the Omani population was observed over the past five decades. Non-communicable diseases (NCDs), like cardiovascular diseases, cancer, and diabetes, became more prevalent compared to infectious diseases [19]. This change can be attributed to multiple factors, such as (1) behavioral factors: sedentary lifestyle, low-quality diet, and tobacco and alcohol use, (2) biological/metabolic factors: overweight/obesity, high blood pressure, hyperglycemia, and hyperlipidemia; and (3) environmental factors: globalization, urbanization, and socioeconomic status [24,25,26]. In 2022, NCD progress monitors reported that 80% (13,000) of deaths in Oman were from NCDs, with a 21% probability of premature death from NCDs [27]. These numbers are alarming and call for immediate interventions and policy changes to mitigate the rapid increase in NCDs in Oman.

To understand the influence of diet on NCDs in general, Western countries, including the United States (US) [28,29], the United Kingdom (UK) [30], and Canada [31,32], employ validated FFQs for this purpose. Some studies in the Gulf region, such as Kuwait [33,34], United Arab Emirates (UAE) [34], Qatar [35], and Saudi Arabia (SA) [36], have also developed and validated FFQs to assess dietary intake. However, there is no reliable or valid FFQ developed specifically for Omani adults. FFQs are population specific and need to be adapted to accurately represent the population being studied [37].

The prevalence of NCDs related to nutritional factors is high in Oman. Although Oman has had food-based dietary guidelines since 2009, dietary intake has not been evaluated systematically. A culturally specific FFQ would enable population-specific nutritional research, public health surveillance, and evidence-based interventions addressing risk factors of NCDs in Oman. To the best of our knowledge, this study is the first to develop a culturally specific food frequency questionnaire for Omani adults and assess its validity and reliability.

## 2. Materials and Methods

### 2.1. Ethical Approval

This study was reviewed and approved by the Office of the Deputy Vice Chancellor for Postgraduate Studies and Research at Sultan Qaboos University on 18 October 2023. Permission was not needed to adapt the DHQ II to the Omani culture, as it was clearly stated on the National Cancer Institute’s website that the DHQ can be used for research purposes without needing permission. “The Diet History Questionnaire (DHQ) is a freely available food frequency questionnaire (FFQ) developed by staff at the Risk Factor Assessment Branch (RFAB). It can be used by researchers, clinicians, or teachers without permission” [38].

### 2.2. Development of the Food Frequency Questionnaire

#### 2.2.1. Modifications to the Food Frequency Questionnaire

The Omani Food Frequency Questionnaire (OFFQ) was adapted from the Diet History Questionnaire II (DHQ II) developed by the National Cancer Institute [38]. Several criteria guided the adaptation, including cultural relevance and norms, religious norms, commonality of items, availability in the local market, and lifestyle trends (diet and health). Initially, a graduate student and a research assistant, majoring in nutrition, independently reviewed the items on the DHQ II for cultural and religious dietary relevance. If foods did not align, they were marked, and they regularly met to compare notes and reach an agreement. Next, items were assessed for commonness of consumption among Omani adults. In addition, local popular cookbooks were used to compile foods/dishes for the adaptation. Then, the OFFQ was reviewed by an expert panel and modifications were made based on the recommendations. The OFFQ was then reviewed by the research team, and market research was conducted, as a result, to ensure the availability and comparability of processed items in the local market. Two of the largest supermarket chains and six community food stores (small scale) were the center of market research. In the process, macronutrient comparison of the Omani items were obtained from food composition tables [39,40], Food Data Central [41], and nutritional facts on the food labels to ensure comparability between similar items. The research team then translated the OFFQ from English to Arabic and back translated it to Arabic to ensure accuracy of the translation. A meeting with expert panels in the nutrition field was held to finalize the first draft of the OFFQ. The meeting to revise portion sizes, determine the suitability of some food items (addition and elimination), and draft the OFFQ was then finalized.

The modifications resulted in the addition of 43 new questions and 20 new food items that are commonly consumed in the Omani diet: (1) non-alcoholic malt beverages, (2) rutab, (3) tahini or nuts, (4) mushrooms, (5) coconut milk or cooking cream, (6) machboos, qabooli, or mandi rice, (7) biryani rice, (8) ersiya or madrouba, (9) koshary, (10) harees or jareesh, (11) Spreadable cheese, triangle cheese, or cream cheese, (12) thareed, (13) quail, (14) dried fish (malih, kasha, awal), (15) laban up, (16) luqaimat and balah al sham, (17) rahash and baklava, (18) croissants, lolaa, mandazi, maamoul, asida, and khabees, (19) pistachio or other nut cakes, and (20) Omani halwa.

In contrast, food items that are rarely consumed or contradict the Islamic religion were omitted. Thus, questions inquiring about pork were removed. However, questions about alcoholic beverages were retained for their availability and for international comparability of the questionnaire. In total, 39 questions were completely removed, which resulted in the specific omission of 18 food items from the DHQ II: (1) half and half, (2) applesauce, (3) grapefruit, (4) asparagus, (5) potato salad, (6) macaroni and cheese, (7) luncheon or deli-style ham (8) roast turkey, turkey cutlets, or turkey nuggets, (9) baked ham or ham steak, (10) pork (including chops, roasts, and in mixed dishes), (11) gravy, (12) corn bread or corn muffins, (13) biscuits, (14) fruit crisp, cobbler, or strudel, (15) fruit pie (such as apple, blueberry, others), (16) cream, pudding, custard, or meringue pie, (17) pumpkin or sweet potato pie, and (18) pecan pie.

Items in the DHQ II that were not commonly available or consumed in the Omani culture were replaced. For instance, bagels or English muffins were replaced with paratha, chapati, or marduf bread. Another example is ground chicken or turkey, which was replaced with chicken burger, chicken kebab, or minced chicken. Minor modifications were made to the portion sizes; the imperial measurements were converted into the metric system since they are not used in Oman (e.g., ounces were converted into cups). Moreover, questions that included US brands that were not available on the market were replaced with similar Omani name brands.

#### 2.2.2. Face and Content Validity

The face and content validity were assessed using an experience-based approach through expert panels composed of two dietitians with clinical and academic experience, three academics specialized in nutrition, one research assistant, and a graduate student specialized in nutrition. Members were given the draft OFFQ and independently assessed the items. Every meeting was designated for a specific group of items to be reviewed before a meeting. Roundtable group discussions were conducted for discussion. The suitability or lack of suitability of items was established when members unanimously agreed or by the majority. Accordingly, food items found to be irrelevant were omitted, while food items consumed regularly by the majority in Oman were added to the FFQ or retained or partially modified. Moreover, current food trends were added to the FFQ. Over five independent reviews and panel discussions were held to review the adapted FFQ comprehensively. In the process, additional market research was conducted to ensure the availability of specific food items or brands on the local market. Then, two separate experts, also specializing in nutrition, who were not involved in the development process were given paper-based copies of the OFFQ and given one week to provide feedback on the content of the questionnaire. The feedback was then discussed in panel discussion to review the comments.

#### 2.2.3. Pilot Testing

The first draft of the OFFQ was piloted on a group of students and employees (*n* = 13) at SQU who had no idea about the developed questionnaire to assess the inclusiveness of the questionnaire, clarity, and readability. Table 1 summarizes the characteristics of the pilot participants. Each participant was given a paper-based OFFQ to complete. They were verbally instructed to complete the OFFQ within one sitting and to keep a record of when they started and completed it. Feedback was sought from each participant when the FFQs were returned; the process took about 15–20 min of discussion. All participants noted that the OFFQ was comprehensive. Most noted the need to add Haloumi cheese, tea biscuits, and Tang formulas. A final expert panel discussion was conducted to review the outcomes of the pilot testing and the OFFQ to finalize the questionnaire.

#### 2.2.4. Components of the Food Frequency Questionnaire

The final draft of the OFFQ contained 136 food items, 8 dietary supplement questions, and 9 questions on the sociodemographic information, with a total of 415 questions. The questions related to dietary intake were divided into 15 food groups: (1) beverages, (2) fruits, (3) vegetables, potatoes, and dried beans, (4) soups and Middle Eastern foods, (5) rice, pasta, and pizza, (6) cereal, qaroos, and breads, (7) date molasses, jam, and peanut butter, (8) mashakik, shiwa, and cold cuts, (9) meat, poultry, and fish (10), eggs and meat alternatives, (11) chips, rusk, and other snacks, (12) yoghurt and cheese, (13) sweets, baked goods, and desserts, (14) spreads and dressings, and (15) vitamins and supplements.

Similar to the original DHQ II, the OFFQ consisted of two main question types, one about the frequency of consumption over the past month, and another about portion sizes for each food item. Some questions contained additional questions about cooking methods, the addition of fats and dressings or others (add-ons). Response options for consumption frequency included per month, per week, or per day for each food items or a group of food items with similar nutritional content. For instance, cantaloupe, mango, and papaya. Frequency options were provided to estimate the intake frequency, which ranged from eight to nine responses. The eight frequency response categories pertained to all food items except beverages, which were presented as follows: (1 time in the past month, 2–3 times in the past month, 1 time per week, 2 times per week, 3–4 times per week, 5–6 times per week, 1 time per day, 2 or more times per day). Nine frequency options were for beverages ranging from: (1 time in the past month, 2–3 times in the past month, 1–2 times per week, 3–4 times per week, 5–6 times per week, 1 time per day, 2–3 times per day, 4–5 times per day, 6 or more times per day). For portion size estimation, three choices were offered, which were reported in terms of common household items, including teaspoons, tablespoons, and cups. Both intake frequency and portion sizes were based on the original DHQ II.

The OFFQ was deployed in Arabic only since the intended population language is Arabic. The final draft of the OFFQ was converted to an electronic format by Data Mining (DM), https://eqp.datamining.om/ (accessed on 13 August 2024), an Omani company providing various information technology solutions. The OFFQ was hosted on the “Estebyan” platform of the DM [42].

### 2.3. Reliability Study

#### 2.3.1. Study Population and Sample Size

The study targeted adult Omani aged 18–65 years who are part of SQU and the Sultan Qaboos University Hospital (SQUH). Based on the 2022 annual report, the number of employees at SQU and SQUH was 5570; and there were 18,855 students (Sultan Qaboos University, 2024). Thus, the population size was determined as 24,445. Using an online sample size calculator (Raosoft, http://www.raosoft.com/samplesize.html, accessed on 20 January 2023), the proposed sample size was 379 students and employees, taking into account a 95% confidence level and 5% margin of error. A convenience sample of Omani adults (*n* = 62) was obtained from Sultan Qaboos University students and employees.

#### 2.3.2. Recruitment and Screening Process

**Figure 1:**
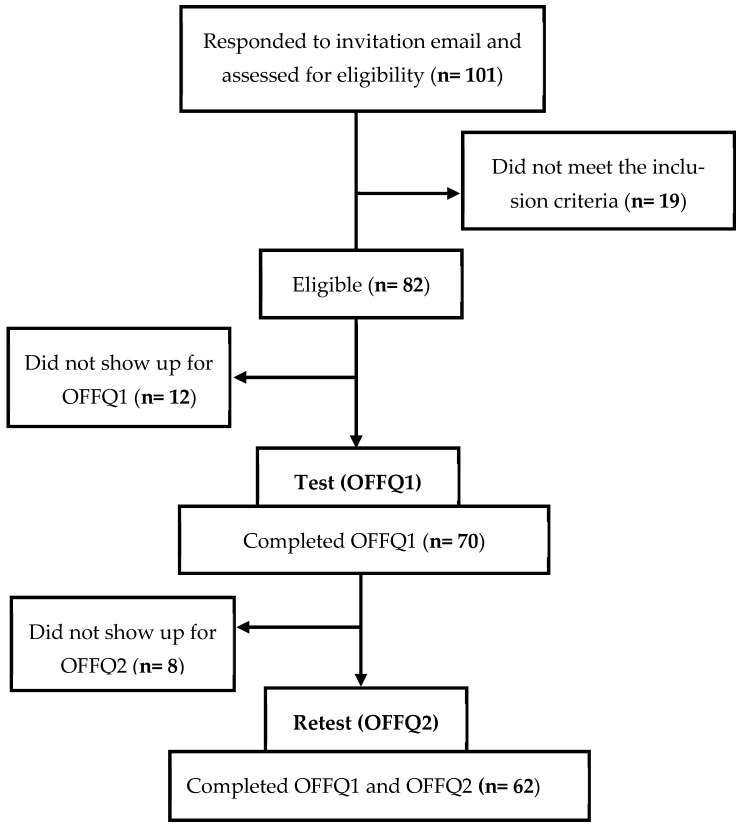
Sample size flow in the study.

An invitation email was deployed to the university students and staff, with a cell phone contact number and an email address for inquiries and expressions of interest. Participants who signed up were contacted and briefed about the study and checked for eligibility before they were formally enrolled in the study. Those enrolled were given choices of set time slots to choose a suitable time to respond to the questionnaire in-person. Figure 1 shows the sample size flow in the study.

#### 2.3.3. Inclusion and Exclusion Criteria

The inclusion criteria were Omani adults aged between 18–65, students or employees at Sultan Qaboos University, not having any plans to travel during the next 2 weeks, no significant change in weight during the last 3 months, and have the mental and physical capacity to provide informed consent for participation in the study. While the exclusion criteria were previously diagnosed with any chronic diseases, depression, inflammatory bowel diseases (IBDs), or eating disorders, undergoing treatment, pregnant or lactating women, and women who gave birth 3 months prior to the study.

#### 2.3.4. Procedure for Data Collection

Test–retest reliability was assessed by administering the OFFQ twice (OFFQ1 and OFFQ2). The second administration of the OFFQ was one to two weeks after the first. The interval was selected to minimize changes in the dietary pattern, as longer intervals could result in underestimation, whereas shorter intervals might lead to overestimation because participants could remember their previous responses [43]. Eligible candidates (*n* = 82) were scheduled to take the first in-person questionnaire (OFFQ1).

The in-person meetings were conducted in a computer laboratory at SQU. The researcher explained how the OFFQ must be completed. Participants were then allowed to ask questions regarding the OFFQ, and the electronic OFFQ1 was administered after that. After the participants completed OFFQ1, a suitable time to take OFFQ2 was arranged. Participants were contacted two days prior to their second meeting as a reminder. During the second meeting, the participants completed OFFQ2. A total of 62 participants completed the two administrations of the OFFQs (OFFQ1 and OFFQ2). The majority of participants took 45 min to 1 h to complete the OFFQ. Study participants were assigned a unique ID number, which was then used to validate participants who completed both OFFQ administrations.

### 2.4. Data Management

A total of 415 questions in the OFFQ were coded by DM; these are only question codes related to the DM platform and the food groupings. For accuracy and data quality, two research members reviewed all question codes with the DM before the reliability study. Four trial tests of the online questionnaire were done by the researchers and reviewed any setting and coding errors before data collection. Responses to items are set as required for all questions with a skip pattern when the consumption had a frequency of “Never” for intake or “No” for items related to the use of nutrient supplements.

The DM provided the research team with the raw data in an Excel file. Participants who only completed OFFQ1 were eliminated from the analyses. Frequency responses were coded numerically starting from 1 depending on the number of response options in a question. For instance, options for the question “Over the past month, how often did you drink carrot juice?” were coded as follows: Never, go to question (2), as 1; 1 time in the past month as 2; 2–3 times in the past month as 3; 1–2 times per week as 4; 3–4 times per week as 5; 5–6 times per week as 6; 1 time per day as 7; 2–3 times per day as 8; 4–5 times per day as 9; 6 or more times per day as 10. Portion responses were coded numerically from 1–3. For instance, options for the question “Each time you drank carrot juice, how much did you usually drink?” were coded as follows: Less than ½ cup as 1; ½ to 1¼ cups as 2; More than 1¼ cups as 3. Thus, participants who did not consume carrot juice at least one time in the past month chose (Never, go to question (2), did not get asked about the portion size of the item. A coding key was created that contained the question code, question number, variable description, and food groups. All data were managed and coded in Excel using the IF function. Responses related to age, weight, and height were categorized into age and BMI groups. BMI was calculated as weight (kg)/height (m^2^).

### 2.5. Statistical Analysis

Statistical analyses were performed using Statistical Package for Social Sciences (SPSS) statistical software version 29.0.2.0. Frequency and percentage were applied to present sociodemographic characteristics. Dietary intake was analyzed based on grouping items into their inherent food groups. Weighted Kappa (K_W_) with linear weights was used for ordinal data to assess the test–retest reliability of each frequency and portion food item in the OFFQ. In addition, a two-way mixed-effects model intraclass correlation coefficient (ICC) with absolute agreement was used to assess the reliability between the test and retest across all food groups. The internal consistency for each food group was assessed using Cronbach’s α. The correlation was considered to be statistically significant when the *p* value was less than 0.05.

## 3. Results

A total of 62 participants completed the OFFQ at two intervals, test and retest. Median weighted Kappa (K_W_) and intraclass correlation coefficient (ICC) are shown to compare the findings of the test–retest reliability for intake frequency and portion sizes. Findings for individual food items are given in Supplementary tables. The internal consistency of the reported intake frequency of food groups and portion sizes at the two intervals are also presented.

### 3.1. Characteristics of the Reliability Study Sample

In total, 62 participants completed both OFFQs (OFFQ1 and OFFQ2) with the mean age for participants being 22.47 years (ranging from 18–40 years). Most participants were students (87.1%, *n* = 54). Gender discretion was similar, with 48.4% (*n* = 30) being male and 51.6% (*n* = 32) being female. More than one-third of the sample were either overweight or obese (BMI ≥ 25 kg/m^2^). Table 2 details the characteristics of the reliability study participants.

### 3.2. Test–Retest Reliability

Table 3 reports the median weighted Kappa (K_W_) and intraclass correlation coefficient (ICC) for all food groups frequency items. The highest K_W_ and ICC values reported (soups and Middle Eastern foods) were 0.601 and 0.807, respectively, while the lowest K_W_ and ICC values reported were 0.395 (Soups and Middle Eastern foods) and 0.569 (eggs and meat alternatives), respectively.

Table 4 reports the median weighted Kappa (K_W_) and intraclass correlation coefficient (ICC) for all food group portion items. The highest K_W_ and ICC values reported (soups and Middle Eastern foods) were 0.580 and 0.821, respectively, while the lowest K_W_ and ICC values reported (yoghurt and cheese) were 0.259 and 0.395, respectively.

### 3.3. Internal Consistency

Of the 15 food groups in the OFFQ, two groups were excluded from the analyses (spreads and dressings and vitamins and supplements). The two groups had very low reported intake and, hence, were not analyzable. The internal consistency of food groups was measured using Cronbach’s α (Table 5). The internal validity, on both the test and retest, of only five food groups had Cronbach’s α > 0.70. Eight food groups had Cronbach’s α < 0.70, ranging from 0.330 to 0.863 for the test and 0.267 to 0.871 for the retest. The results of the group eggs and meat alternatives were not analyzable, as there were few frequency items (two items) related to this group.

As shown in Table 6, Cronbach’s α in both test and retest for only five food groups was reported with Cronbach’s α value lower than <0.70 ranging from 0.404 to 0.731 for the test and ranging from 0.286 to 0.955 for the retest. The remaining food groups were not analyzed since there were too few cases for the analysis. The absence of Cronbach’s α values for some portion items are due to participants reporting “Never” in the intake frequency of the item.

## 4. Discussion

The OFFQ yielded good findings. The median (Kw) in our study showed that the OFFQ had moderate to good test–retest reliability, with the majority of food items within food groups showing moderate to good reliability except in three groups (mashakik.., eggs.., sweets..) for frequency items, and four groups (cereal.., mashakik.., yoghurt.., sweets..) for portion items. On the other hand, the median ICC of food groups showed good agreement in all frequency items except in two groups (mashakik.., eggs..), and in all group portion items except in one group (yoghurt..). The internal consistency of four food groups was ≥0.7 in frequency items while only one in portions items. A number of items did not have calculated Cronbach’s α in portion items because participants reported “Never” in related frequency items.

The assessment of dietary patterns can help determine diet-related risk factors that contribute to the increased prevalence of NCDs. FFQs are used to assess the dietary intake of large populations, as they are considered cost efficient and less burdensome compared to other methods, such as 24-HDRs and WFRs [44]. Thus, FFQs are considered to be the most suitable tool to assess dietary intake in large-scale studies [45]. FFQs need to be comprehensive to capture habitual dietary patterns effectively and assess their relations to NCDs [37], and for that, they contain a large number of items [37]. The newly developed OFFQ contained 136 food items and 8 dietary supplement questions that were methodically added, covering most if not all foods consumed by Omani adults. The number of items in our FFQs is similar to that of Qatar and Malaysia, 153 and 203 food items, respectively [35,46]. FFQs from Western countries are also known to have large numbers of items. One from the USA has 156 items [29], and another from the New Zealand has 154 items [47].

The OFFQ was assessed for reliability with one to two week intervals between two administrations. The interval between the two administrations is an important factor that could affect the reliability of a tool [46]. Long intervals may lead to underestimations and dietary intake may change during that time. Short intervals may result in overestimation as participants may be able to recall their answers for the first administration [43]. Other studies had varied intervals between the two administrations ranging from one month to a year [30,32].

Previous studies reported ICC values for food groups among different ethnic groups, ranging from 0.33 to 0.73 in South Asians and 0.30 to 0.83 in Europeans [48]. A study in China reported a median ICC for food groups between the two FFQ administrations ranging from 0.26 to 0.65. However, our study reported higher median ICC values, ranging from 0.569 to 0.807 for frequency items and ranging from 0.395 to 0.821 for portion items. The higher ICC values reported in our study could be attributed to the duration between the two OFFQ administrations while the interval between the two administrations in the Chaina study was nine months [49], which is a long interval.

The median K_W_ values for most frequency (*n* = 10) and portions (*n* = 9) groups show moderate agreement [50]. A closer look at individual food items of frequency and portions show variable agreement. For example, the weighted Kappa values for the majority of items (*n* = 16) in beverage frequency and portions (Tables S1 and S2) showed moderate to very good agreement [50]. All frequency items in the “soups and Middle Eastern foods” group showed moderate to good agreement (Table S7) [50]. The portion items in this group showed fair, moderate to good agreement (Table S8) [50]. However, there are only three items in this group. The majority of validation and reliability studies compared nutrients and energy intakes [35,51,52]. In the current study, nutrient analyses were not done; thus, comparing our results with previous studies should be viewed with caution.

Across the frequency of all 13 analyzed food groups (excluding two food groups: “spreads and dressings” and “vitamins and supplements”), one item (sport drinks) had the highest agreement (K_W_ = 0.721; Table S1) and another item (macaroni salad) had the lowest agreement (K_W_ = 0.030; Table S9). A similar study assessing the reliability of a short FFQ among children in Australia reported having the highest reliability (K_W_ = 0.85) for take-away foods but the lowest reliability (K_W_ = 0.37) for red meat [53]. In another study, K_W_ values showed fair to moderate agreement for micronutrients (0.3–0.4) and macronutrients (0.5–0.6) [54]. Although our study did not perform nutrient analysis, K_W_ values for the majority of food items ranged from moderate to good agreement. For portion items across all the groups (excluding the two groups above), one item (other fish that was not fired) had the lowest agreement (K_W_ = 0.029; Table S18) and two items (energy drinks, Table S2; pistachio or other nut cake, Table S26) had the highest agreement (K_W_ = 1). No studies were found to have reported agreement based on portion sizes to compare our findings with.

Weighted Kappa values for portions tended to be lower than those for frequency items withing the same group. The agreement, K_W_ values, for frequency items in the “yoghurt and cheese” group showed fair to moderate agreement (Table S23) [50]. However, the K_W_ values for portion items of the same group showed poor to fair agreement (Table S24) [50]. Another example is the K_W_ values for frequency items in the “vegetables, potatoes and dried beans” group. The majority (*n* = 22/28) of items showed moderate to good agreement, ranging from K_W_ = 0.290 for string beans, green beans, or okra to K_W_ = 0.676 for sweet peppers (Table S5) [50]. The agreement for each portion item in this group showed fair to moderate agreement, while cooked greens and broccoli showed poor agreement (Table S6) [50]. In the FFQ for the Mediterranean diet using nutrients, the highest and the lowest weighted Kappa agreements were 0.32 and 0.63 for cholesterol and protein, respectively, among all assessed nutrients [55]. In our study the median K_W_ between the two OFFQ administrations showed fair to moderate agreement [50] with K_W_ values from 0.379 to 0.601 for frequency items, K_W_ values from 0.259 to 0.580 for portion items between the two OFFQ administrations.

Overall, five frequency groups showed moderate to good agreement: (1) beverages, (2) fruits, (3) vegetables, potatoes, and dried beans, (4) soups and Middle Eastern foods, and (5) rice, pasta, pizza. While three portion groups showed moderate to good agreement: (1) fruits, (2) rice, pasta, and pizza, and (3) meat, poultry, and fish. Moreover, two portion items showed moderate to very good agreement: (1) beverages and (2) meat, poultry, and fish. Moderate agreement was reported across two frequency items: (1) date molasses, jam, and peanut butter and (2) chips, rusk, and other snacks. Similarly, moderate agreement was reported across two portion items: (1) date molasses, jam, and peanut butter and (2) eggs and meat alternatives.

Weighted Kappa and ICC values for some food items showed consistent results. This confirms the reliability of the OFFQ and shows that, irrespective of the statistical method employed, there is an alignment between the two methods (i.e., K_W_ and ICC). There was an observed slight increase in ICC values in OFFQ1 compared to OFFQ2. This could be attributed to the engagement level and attention of the participants while completing the OFFQ1. Similar studies have also reported higher ICC values in the test FFQ compared to the retest FFQ [52,56]. The confidence intervals for most frequency and portion items are mostly small, indicating good confidence in results.

A range of food groups had good reliability [57] with Cronbach’s α > 0.70. However, most food groups had a Cronbach’s α value < 0.70. A FFQ reliability study in Brunei reported Cronbach’s α values ranging from 0.505 to 0.986 with most values being >0.900 [58]. Similarly, our study reported Cronbach’s α values for intake frequency ranging from 0.330 to 0.863 for OFFQ1 and values ranging from 0.267 to 0.871 for OFFQ2. While Cronbach’s α for portion items ranged from 0.404 to 0.603 for OFFQ1 and ranging from 0.286 to 0.593 for OFFQ2. In a short FFQ estimating cholesterol intake among Indonesians, Cronbach’s α was 0.757 [59].

Although our sample size (*n* = 62) was greater than the minimum recommendation of sample size for reliability studies, which is 30 participants [15]. A larger sample size could have provided more reliable results. However, a sample size of at least 50–100 participants would suffice [60]. The study sample had participants from 8 out of the 11 governances in Oman. Moreover, due to the length of the questionnaire, which contains questions about frequency, portion, and subsequent questions that inquired about fat consumption and cooking methods, this could have led to higher variation in responses, thus affecting the reliability of the questionnaire [61,62]. Also, participants may have experienced fatigue and boredom due to the length of the questionnaire, which may have led to underreporting [37].

While high intraclass correlation coefficients (ICC) or Kappa values are generally interpreted as indicators of good reliability, they may sometimes reflect consistent but biased recall or homogeneity in responses rather than true dietary accuracy. For instance, participants might consistently misreport intake or rely on similar recall strategies, which can inflate reliability without ensuring validity.

This study presented several strengths. Most importantly, it addresses the gap in literature by developing the first reliable and valid semi-quantitative FFQ in Oman specifically for adults. The development of a reliable FFQ will enable the assessment of the relationship between diet and disease in future nutritional epidemiological studies. The OFFQ was adapted from DHQ II developed by the National Cancer Institute. Generally, the DHQ was used in many studies [35,51,52,63]. Three studies have validated DHQ I, which showed that DHQ I can accurately estimate nutrient intakes. However, DHQ II was not validated since minimal changes were introduced thus no major changes in the validation are low [38]. In order to develop a culturally specific FFQ for Omani adults, traditional Omani dishes were added carefully to the OFFQ. In contract, Western foods were removed only if they were rarely consumed among Omani adults. The structure of the OFFQ was similar to the DHQ II.

The FFQ can be applied in national nutrition surveillance to monitor dietary trends, nutrient intake, and food consumption patterns in large populations, informing public health policies and dietary guidelines. Moreover, FFQs are commonly used in large cohort studies to explore associations between diet and chronic diseases, such as cardiovascular disease, cancer, and diabetes. For future studies, the FFQ can be validated against biological markers such as urinary nitrogen, to assess the relative validity of the tool. Food records and 24-HDRs can also be used to assess the relative validity of the FFQ; however, multiple re-calls or food records are needed.

## 5. Conclusions

Overall, the results of our study showed that the newly developed OFFQ had moderate to good test–retest reliability for most food items and food groups. This is the first study in Oman to develop a comprehensive and validated instrument for assessing Omani dietary intake that includes both traditional foods as well as Western foods. The newly developed OFFQ can be used to monitor and quantify dietary pattens among Omani adults. In addition, it can be used to understand the relationship between diet and disease in epidemiological studies. Nevertheless, further validation and reliability studies are required as this is the first attempt in Oman to develop a valid and reliable tool that can accurately measure dietary intake.

The study has several limitations, and caution is warranted when interpreting the findings. These include a small sample size, a semi-homogeneous sample with the majority being university students, the absence of relative validity assessment, and a relatively high level of education. Also, the number of food items in the questionnaire could have affected the accuracy of estimating the dietary intake; however, this is an inherent characteristic of such tools. Long food lists may lead to over estimation as more choices are presented for the participants [64], while shorter lists may limit their options, leading to the underestimation of food intake [65]. Consistently, two group items (“spreads and dressings” and “vitamins and supplements”) had few cases to be analyzed due to low reported intake frequency, and they were not added to tables.

Future research warrants the application of the OFFQ to larger and diverse samples of individuals from the community; the assessment of the relative validity of the OFFQ by comparing its correlation to 24-h dietary recall or weighted food records; and energy and nutrient analyses of micronutrients and macronutrients.

## Figures and Tables

**Table 1:** Characteristics of participants in the pilot study (*n* = 13).

Variable	*n* (%)
Gender	
Male	3 (23%)
Female	10 (77%)
Age (years)	
18–24	8 (61.5%)
≥25	5 (38.5%)
Permanent Residency (Governorate)	
Al Dakhiliyah	2 (15.4%)
Al Dhahirah	1 (7.7%)
North Al Batinah	1 (7.7%)
South Al Batinah	3 (23%)
Musandam	1 (7.7%)
Muscat	3 (23%)
North Al Sharqiyah	2 (15.4%)
Employment status	
Employee	5 (38.5%)
Student	8 (61.5%)
Highest level of education	
High school	7 (53.8%)
Postgraduate degree	6 (46.2%)
Marital status	
Married	4 (30.8%)
Unmarried	9 (69.2%)
Individual monthly income (OMR)	
Less than 500	7 (53.8%)
More than 500	6 (46.2%)

OMR (Omani Rial). 1 OMR ≈ USD 2.6 (pegged).

**Table 2:** Characteristics of participants in the test–retest of the FFQ (*n* = 62).

Variable	*n* (%)
Gender	
Male	30 (48.4%)
Female	32 (51.6%)
Age (years)	
18–24	50 (80.6%)
≥25	12 (19.4%)
Permanent Residency (Governorate)	
Al Dakhiliyah	13 (21%)
Al Dhahirah	7 (11.3%)
North Al Batinah	7 (11.3%)
South Al Batinah	9 (14.5%)
Musandam	1 (1.6%)
Al Buraymi	1 (1.6%)
Muscat	21 (33.9%)
Dhofar	3 (4.8%)
Employment status	
Employee	8 (12.9%)
Student	54 (87.1%)
Highest level of education	
High school	45 (72.6%)
Post-secondary diploma	4 (6.5%)
Postgraduate degree	13 (21%)
Marital status	
Married	4 (6.5%)
Unmarried	58 (93.5%)
Individual monthly income (OMR)	
No source of income	31 (50%)
Less than 500	22 (35.5%)
More than 500	9 (14.5%)
BMI (kg/m^2^)	
<25	40 (64.5%)
25–29.9	15 (24.2%)
≥30	7 (11.3%)

**Table 3:** Comparison of frequency median intakes of food groups between OFFQ1 and OFFQ2.

Food Group	K_W_^a^	ICC ^b^
Beverages	0.545	0.725
Fruits	0.548	0.803
Vegetables, potatoes, dried beans	0.488	0.745
Soups, Middle Eastern foods	0.601	0.807
Rice, pasta, pizza	0.476	0.705
Cereal, qaroos, breads	0.425	0.709
Date molasses, jam, peanut butter	0.513	0.735
Mashakik, shiwa, cold cuts	0.379	0.576
Meat, poultry, fish	0.468	0.689
Eggs, meat alternatives	0.398	0.569
Chips, rusk, other snacks	0.524	0.773
Yoghurt, cheese	0.427	0.716
Sweets, baked goods, desserts	0.395	0.695

^a^ Weighted Kappa. ^b^ Intraclass correlation coefficient.

**Table 4:** Comparison of portion median intakes of food groups between the OFFQ1 and OFFQ2.

Food Group	K_W_^a^	ICC ^b^
Beverages	0.500	0.692
Fruits	0.429	0.696
Vegetables, potatoes, dried beans	0.422	0.639
Soups, Middle Eastern foods	0.580	0.821
Rice, pasta, pizza	0.432	0.656
Cereal, qaroos, breads	0.401	0.639
Date molasses, jam, peanut butter	0.462	0.728
Mashakik, shiwa, cold cuts	0.403	0.713
Meat, poultry, fish	0.481	0.727
Eggs, meat alternatives	0.551	0.793
Chips, rusk, other snacks	0.489	0.697
Yoghurt, cheese	0.259	0.395
Sweets, baked goods, desserts	0.402	0.621

^a^ Weighted Kappa. ^b^ Intraclass correlation coefficient.

**Table 5:** Internal consistency of intake frequency of the OFFQ food groups.

	Test	Retest
Food Group	Cronbach’s α	Cronbach’s α
Beverages	0.386	0.502
Fruits	0.789	0.798
Vegetables, potatoes, dried beans	0.863	0.871
Soups, Middle Eastern foods	0.390	0.267
Rice, pasta, pizza	0.500	0.484
Cereal, qaroos, breads	0.560	0.516
Date molasses, jam, peanut butter	0.330	0.414
Mashakik, shiwa, cold cuts	0.502	0.347
Meat, poultry, fish	0.700	0.751
Eggs, meat alternatives	-	-
Chips, rusk, other snacks	0.568	0.633
Yoghurt, cheese	0.455	0.293
Sweets, baked goods, desserts	0.744	0.720

**Table 6:** Internal consistency of the FFQ food group portion sizes at the test and retest.

	Test	Retest
Food Group	Cronbach’s α	Cronbach’s α
Beverages	-	-
Fruits	-	-
Vegetables, potatoes, dried beans	-	-
Soups, Middle Eastern foods	0.603	0.593
Rice, pasta, pizza	-	0.810
Cereal, qaroos, breads	0.731	0.955
Date molasses, jam, peanut butter	0.520	0.272
Mashakik, shiwa, cold cuts	0.645	0.538
Meat, poultry, fish	-	-
Eggs, meat alternatives	-	-
Chips, rusk, other snacks	-	-
Yoghurt, cheese	0.404	0.286
Sweets, baked goods, desserts	-	-

## Data Availability

The data presented in this study are available on request from the corresponding author due to the privacy of participants.

## Supplementary Materials

The following supporting information can be downloaded at: https://www.mdpi.com/article/10.3390/nu17132220/s1, Table S1. Test–retest reliability of intake frequency of the beverages group; Table S2. Test–retest reliability of the intake portion of the beverages group; Table S3. Test–retest reliability of the intake frequency of the fruits group; Table S4. Test–retest reliability of the intake portion of the fruits group; Table S5: Test–retest reliability of the intake frequency of the vegetables, potatoes, and dried beans group; Table S6: Test–retest reliability of the intake portion of the vegetables, potatoes, and dried beans group; Table S7: Test–retest reliability of the intake frequency of the soups and Middle Eastern foods group; Table S8: Test–retest reliability of the intake portion of the soups and Middle Eastern foods group; Table S9: Test–retest reliability of the intake frequency of the rice, pasta, and pizza group; Table S10: Test–retest reliability of the intake portion of the rice, pasta, and pizza group; Table S11: Test–retest reliability of the intake frequency of the cereal, qaroos, and breads group; Table S12: Test–retest reliability of the intake portion of the cereal, qaroos, and breads group; Table S13: Test–retest reliability of the intake frequency of the date molasses, jam, and peanut butter group; Table S14: Test–retest reliability of the intake portion of the date molasses, jam, and peanut butter group; Table S15: Test–retest reliability of the intake frequency of the mashakik, shiwa, and cold cuts group; Table S16: Test–retest reliability of the intake portion of the mashakik, shiwa, and cold cuts group; Table S17: Test–retest reliability of the intake frequency of the meat, poultry, and fish group; Table S18: Test–retest reliability of the intake portion of the meat, poultry, and fish group; Table S19: Test–retest reliability of the intake frequency of the eggs and meat alternatives group; Table S20: Test–retest reliability of the intake portion of the eggs and meat alternatives group; Table S21: Test–retest reliability of the intake frequency of the chips, rusk, and other snacks group; Table S22: Test–retest reliability of the intake portion of the chips, rusk, and other snacks group; Table S23: Test–retest reliability of the intake frequency of the yoghurt and cheese group; Table S24: Test–retest reliability of the intake portion of the yoghurt and cheese group; Table S25: Test–retest reliability of the intake frequency of the sweets, baked goods, and desserts group; Table S26: Test–retest reliability of the intake portion of the sweets, baked goods, and desserts group.

## References

[1] Alkerwi A. (2014). Diet quality concept. Nutrition.

[2] Wirt A., Collins C.E. (2009). Diet quality–what is it and does it matter?. Public Health Nutr..

[3] McNaughton S.A. (2010). Dietary patterns and diet quality: Approaches to assessing complex exposures in nutrition. Australas. Epidemiol..

[4] Angelopoulos P., Kourlaba G., Kondaki K., Fragiadakis G.A., Manios Y. (2009). Assessing children’s diet quality in Crete based on Healthy Eating Index: The Children Study. Eur. J. Clin. Nutr..

[5] Hodge A., Bassett J. (2016). What can we learn from dietary pattern analysis?. Public Health Nutr..

[6] Hu F.B. (2002). Dietary pattern analysis: A new direction in nutritional epidemiology. Curr. Opin. Lipidol..

[7] Gerber M. (2001). The comprehensive approach to diet: A critical review. J. Nutr..

[8] Agnoli C., Pounis G., Krogh V. (2019). Dietary pattern analysis. Analysis in Nutrition Research.

[9] Pinto A., Severo M., Oliveira A. (2021). Use of a hybrid method to derive dietary patterns in 7 years olds with explanatory ability of body mass index at age 10. Eur. J. Clin. Nutr..

[10] Ocké M.C. (2013). Evaluation of methodologies for assessing the overall diet: Dietary quality scores and dietary pattern analysis. Proc. Nutr. Soc..

[11] Schulze M.B., Hoffmann K., Kroke A., Boeing H. (2003). An approach to construct simplified measures of dietary patterns from exploratory factor analysis. Br. J. Nutr..

[12] Previdelli Á.N., de Andrade S.C., Fisberg R.M., Marchioni D.M. (2016). Using two different approaches to assess dietary patterns: Hypothesis-driven and data-driven analysis. Nutrients.

[13] Hoffmann K., Schulze M.B., Schienkiewitz A., Nöthlings U., Boeing H. (2004). Application of a new statistical method to derive dietary patterns in nutritional epidemiology. Am. J. Epidemiol..

[14] Hu F.B., Satija A., Rimm E.B., Spiegelman D., Sampson L., Rosner B., Camargo C.A., Stampfer M., Willett W.C. (2016). Diet assessment methods in the nurses’ health studies and contribution to evidence-based nutritional policies and guidelines. Am. J. Public Health.

[15] Willett W., Lenart E. (2013). Reproducibility and validity of food frequency questionnaires. Nutr. Epidemiol..

[16] Walton J. (2015). Dietary assessment methodology for nutritional assessment. Top. Clin. Nutr..

[17] Shim J.-S., Oh K., Kim H.C. (2014). Dietary assessment methods in epidemiologic studies. Epidemiol. Health.

[18] World Health Organization (2024). Obesity and Overweight. https://www.who.int/news-room/fact-sheets/detail/obesity-and-overweight.

[19] Al-Lawati J.A., Mabry R., Mohammed A.J. (2008). Addressing the threat of chronic diseases in Oman. Prev. Chronic Dis..

[20] Al-Riyami A.A., Afifi M.M. (2003). Prevalence and correlates of obesity and central obesity among Omani adults. Saudi Med. J..

[21] Al-Mawali A., Jayapal S.K., Morsi M., Al-Shekaili W., Pinto A.D., Al-Kharusi H., Al-Harrasi A., Al-Balushi Z., Idikula J. (2021). Prevalence of risk factors of non-communicable diseases in the Sultanate of Oman: STEPS survey 2017. PLoS ONE.

[22] Badran M., Laher I. (2011). Obesity in Arabic-speaking countries. J. Obes..

[23] Okati-Aliabad H., Ansari-Moghaddam A., Kargar S., Jabbari N. (2022). Prevalence of obesity and overweight among adults in the Middle East countries from 2000 to 2020: A systematic review and meta-analysis. J. Obes..

[24] Al-Mawali A. (2015). Non-communicable diseases: Shining a light on cardiovascular disease, Oman’s biggest killer. Oman Med. J..

[25] Popkin B.M. (2006). Global nutrition dynamics: The world is shifting rapidly toward a diet linked with noncommunicable diseases. Am. J. Clin. Nutr..

[26] World Health Organization (2023). Noncommunicable Diseases Fact Sheet. https://www.who.int/news-room/fact-sheets/detail/noncommunicable-diseases.

[27] World Health Organization Noncommunicable Diseases: Progress Monitor 2022. https://iris.who.int/handle/10665/353048.

[28] Subar A.F., Thompson F.E., Kipnis V., Midthune D., Hurwitz P., McNutt S., McIntosh A., Rosenfeld S. (2001). Comparative validation of the Block, Willett, and National Cancer Institute food frequency questionnaires: The Eating at America’s Table Study. Am. J. Epidemiol..

[29] Kristal A.R., Kolar A.S., Fisher J.L., Plascak J.J., Stumbo P.J., Weiss R., Paskett E.D. (2014). Evaluation of web-based, self-administered, graphical food frequency questionnaire. J. Acad. Nutr. Diet..

[30] Fallaize R., Forster H., Macready A.L., Walsh M.C., Mathers J.C., Brennan L., Gibney E.R., Gibney M.J., Lovegrove J.A. (2014). Online dietary intake estimation: Reproducibility and validity of the Food4Me food frequency questionnaire against a 4-day weighed food record. J. Med. Internet Res..

[31] Labonté M.-È., Cyr A., Baril-Gravel L., Royer M., Lamarche B. (2012). Validity and reproducibility of a web-based, self-administered food frequency questionnaire. Eur. J. Clin. Nutr..

[32] Liu L., Wang P.P., Roebothan B., Ryan A., Tucker C.S., Colbourne J., Baker N., Cotterchio M., Yi Y., Sun G. (2013). Assessing the validity of a self-administered food-frequency questionnaire (FFQ) in the adult population of Newfoundland and Labrador, Canada. Nutr. J..

[33] Dehghan M., Al-Hamad N., McMillan C.R., Prakash P., Merchant A.T. (2009). Comparison of a semi-quantitative food frequency questionnaire with 24-hour dietary recalls to assess dietary intake of adult Kuwaitis. Saudi Med. J..

[34] Alawadhi B., Fallaize R., Franco R.Z., Hwang F., Lovegrove J. (2021). Web-based dietary intake estimation to assess the reproducibility and relative validity of the eatwellq8 food frequency questionnaire: Validation study. JMIR Form. Res..

[35] Bawadi H., Akasheh R.T., Kerkadi A., Haydar S., Tayyem R., Shi Z. (2021). Validity and reproducibility of a food frequency questionnaire to assess macro and micro-nutrient intake among a convenience cohort of healthy adult Qataris. Nutrients.

[36] Gosadi I.M., Alatar A.A., Otayf M.M., AlJahani D.M., Ghabbani H.M., AlRajban W.A., Alrsheed A.M., Al-Nasser K.A. (2017). Development of a Saudi Food Frequency Questionnaire and testing its reliability and validity. Saudi Med. J..

[37] Cade J., Thompson R., Burley V., Warm D. (2002). Development, validation and utilisation of food-frequency questionnaires–A review. Public Health Nutr..

[38] National Cancer Institute (2023). Background on Diet History Questionnaire II (DHQ-II) for U.S. & Canada. https://epi.grants.cancer.gov/dhq2/about/.

[39] Musaiger A.O. (2006). Food Composition Tables for Arab Gulf Countries (Gulfoods).

[40] Sawaya W.N., Al-Awadi F., Eid N., Dashti B.H. (1998). Food Composition Kuwaiti Composite Dishes.

[41] United States Department of Agriculture (2024). Food Data Central. https://fdc.nal.usda.gov/.

[42] Data Mining (2024). Estebyan. https://eqp.datamining.om/.

[43] Fernández-Ballart J.D., Piñol J.L., Zazpe I., Corella D., Carrasco P., Toledo E., Perez-Bauer M., Martínez-González M.Á., Salas-Salvadó J., Martín-Moreno J.M. (2010). Relative validity of a semi-quantitative food-frequency questionnaire in an elderly Mediterranean population of Spain. Br. J. Nutr..

[44] Collins C.E., Boggess M.M., Watson J.F., Guest M., Duncanson K., Pezdirc K., Rollo M., Hutchesson M.J., Burrows T.L. (2014). Reproducibility and comparative validity of a food frequency questionnaire for Australian adults. Clin. Nutr..

[45] Aoun C., Bou Daher R., El Osta N., Papazian T., Khabbaz L.R. (2019). Reproducibility and relative validity of a food frequency questionnaire to assess dietary intake of adults living in a Mediterranean country. PLoS ONE.

[46] Shahar S., Shahril M.R., Abdullah N., Borhanuddin B., Kamaruddin M.A., Yusuf N.A.M., Dauni A., Rosli H., Zainuddin N.S., Jamal R. (2021). Development and relative validity of a semiquantitative food frequency questionnaire to estimate dietary intake among a multi-ethnic population in the Malaysian Cohort Project. Nutrients.

[47] Sam C.H., Skeaff S., Skidmore P.M. (2014). A comprehensive FFQ developed for use in New Zealand adults: Reliability and validity for nutrient intakes. Public Health Nutr..

[48] Kelemen L.E., Anand S.S., Vuksan V., Yi Q., Teo K.K., Devanesen S., Yusuf S. (2003). Development and evaluation of cultural food frequency questionnaires for South Asians, Chinese, and Europeans in North America. J. Am. Diet. Assoc..

[49] Zhuang M., Yuan Z., Lin L., Hu B., Wang X., Yang Y., Chen X., Jin L., Lu M., Ye W. (2012). Reproducibility and relative validity of a food frequency questionnaire developed for adults in Taizhou, China. PLoS ONE.

[50] McHugh M.L. (2012). Interrater reliability: The kappa statistic. Biochem. Med..

[51] Tayyem R.F., Abu-Mweis S.S., Bawadi H.A., Agraib L., Bani-Hani K. (2014). Validation of a food frequency questionnaire to assess macronutrient and micronutrient intake among Jordanians. J. Acad. Nutr. Diet..

[52] El Kinany K., Garcia-Larsen V., Khalis M., Deoula M.M.S., Benslimane A., Ibrahim A., Benjelloun M.C., El Rhazi K. (2018). Adaptation and validation of a food frequency questionnaire (FFQ) to assess dietary intake in Moroccan adults. Nutr. J..

[53] Flood V.M., Wen L.M., Hardy L.L., Rissel C., Simpson J.M., Baur L.A. (2014). Reliability and validity of a short FFQ for assessing the dietary habits of 2–5-year-old children, Sydney, Australia. Public Health Nutr..

[54] El Sayed Ahmad R., Baroudi M., Shatila H., Nasreddine L., Chokor F.A.Z., Chehab R.F., Forman M.R., Naja F. (2020). Validity and reproducibility of a culture-specific food frequency questionnaire in Lebanon. Nutrients.

[55] Athanasiadou E., Kyrkou C., Fotiou M., Tsakoumaki F., Dimitropoulou A., Polychroniadou E., Menexes G., Athanasiadis A.P., Biliaderis C.G., Michaelidou A.-M. (2016). Development and validation of a Mediterranean oriented culture-specific semi-quantitative food frequency questionnaire. Nutrients.

[56] Marchioni D.M.L., Voci S.M., Lima F.E.L.d., Fisberg R.M., Slater B. (2007). Reproducibility of a food frequency questionnaire for adolescents. Cad. Saude Publica.

[57] Gliem J.A., Gliem R.R. Calculating, interpreting, and reporting Cronbach’s alpha reliability coefficient for Likert-type scales. Proceedings of the Midwest Research-to-Practice Conference in Adult, Continuing, and Community Education.

[58] Marshidi S., Kaur S., Chin K.H., Ulaganathan V., Tarif M. (2024). Development, Validation, and Reproducibility of a Food Frequency Questionnaire (FFQ) for Adult Population in Brunei Darussalam. Malays. J. Med. Health Sci..

[59] Nindya T.S., Mahmudiono T., Rachmah Q. (2021). The estimation of cholesterol intake in elderly: Reliability and validity of short, Semi-Quantitative Food Frequency Questionnaire (SQ-FFQ). J. Nutr. Health.

[60] Cade J.E., Burley V.J., Warm D.L., Thompson R.L., Margetts B.M. (2004). Food-frequency questionnaires: A review of their design, validation and utilisation. Nutr. Res. Rev..

[61] Block G., Hartman A.M. (1989). Issues in reproducibility and validity of dietary studies. Am. J. Clin. Nutr..

[62] Bohlscheid-Thomas S., Hoting I., Boeing H., Wahrendorf J. (1997). Reproducibility and relative validity of food group intake in a food frequency questionnaire developed for the German part of the EPIC project. European Prospective Investigation into Cancer and Nutrition. Int. J. Epidemiol..

[63] Toorang F., Sasanfar B., Jahromi S.R., Koujan S.E., Narmcheshm S., Rafei A., Zendehdel K. (2019). Validation of diet history questionnaire in assessing energy and nutrient intakes of Iranian population. Iran. J. Public Health.

[64] Kowalkowska J., Slowinska M.A., Slowinski D., Dlugosz A., Niedzwiedzka E., Wadolowska L. (2013). Comparison of a full food-frequency questionnaire with the three-day unweighted food records in young Polish adult women: Implications for dietary assessment. Nutrients.

[65] Rockett H.R., Berkey C.S., Colditz G.A. (2007). Comparison of a short food frequency questionnaire with the Youth/Adolescent Questionnaire in the Growing Up Today Study. Int. J. Pediatr. Obes..

