# Supplementary material for: Development, Validity, and Reliability of a Food Frequency Questionnaire for Omani Adults
Source: Nutrients. 2025 Jul 4;17(13):2220. doi: 10.3390/nu17132220 (PMC12251943; doi:10.3390/nu17132220)
Supplement: Supplementary file 1 [file nutrients-17-02220-s001.zip › nutrients-3700891-supplementary.pdf]

Nutrients-3700891: Development, Validity, and Reliability of a Food Frequency Questionnaire for Omani Adults

Supplementary Tables: Weighted Kappa (k_w) and intraclass correlation coefficient (ICCs) for intake frequency and portion size of food items in the Omani Food Frequency Questionnaire (OFFQ)

Table S1. Test–retest reliability of the intake frequency of the beverages group

**Beverages**	**Kw** [a]	**SE**[b]	***p***[c]	**95% CI**[d]	**ICC**[e]	**95% CI**[d]
Carrot juice	0.444	0.156	< 0.001	[0.138-0.750]	0.617	[0.362-0.770]
Vegetable juice	0.387	0.157	< 0.001	[0.078-0.695]	0.604	[0.349-0.760]
100% orange juice	0.557	0.077	< 0.001	[0.407-0.708]	0.837	[0.731-0.902]
100% other fruit juices	0.452	0.082	< 0.001	[0.292-0.612]	0.700	[0.501-0.819]
Fruit drinks	0.424	0.077	< 0.001	[0.273-0.574]	0.721	[0.537-0.832]
Milk as a beverage	0.656	0.058	< 0.001	[0.541-0.770]	0.884	[0.809-0.930]
Flavored milk	0.369	0.089	< 0.001	[0.194-0.543]	0.631	[0.385-0.778]
High-protein beverages	0.385	0.146	< 0.001	[0.099-0.672]	0.686	[0.480-0.810]
Soft drinks	0.711	0.054	< 0.001	[0.606-0.817]	0.925	[0.875-0.955]
Malt drinks (non-alcoholic)	0.545	0.178	< 0.001	[0.197-0.893]	0.725	[0.543-0.835]
Sport drinks	0.721	0.127	< 0.001	[0.473-0.969]	0.823	[0.706-0.893]
Energy drinks	0.549	0.143	< 0.001	[0.269-0.828]	0.790	[0.653-0.873]
Water	0.592	0.095	< 0.001	[0.407-0.778]	0.823	[0.674-0.900]
Median	0.545				0.725	

[a] Weighted Kappa. [b] Standard Error. [c] P-value. [d] Confidence Interval. [e] Intraclass Correlation Coefficient

Table S2. Test–retest reliability of the intake portion of the beverages group

**Beverages**	**Kw** [a]	**SE**[b]	***p***[c]	**95% CI**[d]	**ICC**[e]	**95% CI**[d]
Carrot juice	-	0.375	0.248	[-1.235-0.235]	-	-
Vegetable juice	-	-	-	-	-	-
100% orange juice	0.557	0.077	< 0.001	[0.407-0.708]	0.673	[0.338-0.839]
100% other fruit juices	0.251	0.144	0.046	[-0.031-0.533]	0.471	[-0.082-0.748]
Fruit drinks	0.366	0.140	0.002	[0.092-0.640]	0.521	[0.130-0.736]
Milk as a beverage	0.403	0.145	0.001	[0.120-0.687]	0.665	[0.359-0.824]
Flavored milk	0.475	0.150	0.001	[0.181-0.768]	0.536	[0.011-0.782]
High-protein beverages	0.571	0.353	0.121	[-0.121-1.264]	0.762	[-0.440-0.966]
Soft drinks	0.473	0.123	< 0.001	[0.233-0.714]	0.711	[0.469-0.842]
Malt drinks (non-alcoholic)	0.500	0.375	0.248	[-0.235-1.235]	0.727	[-1.809-0.982]
Sport drinks	0.588	0.347	0.088	[-0.093-1.269]	0.769	[-0.189-0.960]
Energy drinks	1.000	0.000	0.083	[1.000-1.000]	1.000	-
Water	0.453	0.103	< 0.001	[0.252-0.655]	0.632	[0.373-0.784]
Coffee	0.592	0.075	< 0.001	[0.446-0.739]	0.813	[0.691-0.887]
Iced tea	0.574	0.078	< 0.001	[0.421-0.727]	0.816	[0.696-0.889]
Hot tea	0.384	0.078	< 0.001	[0.231-0.536]	0.655	[0.430-0.791]
Median	0.500				0.692	

[a] Weighted Kappa. [b] Standard Error. [c] P-value. [d] Confidence Interval. [e] Intraclass Correlation Coefficient

Table S3. Test–retest reliability of the intake frequency of the fruits group

Fruits	Kw [a]	SE[b]	*p*[c]	95% CI[d]	ICC[e]	ICC[e]
Rutab	0.679	0.095	< 0.001	[0.492-0.866]	0.861	[0.770-0.916]
Apples	0.609	0.066	< 0.001	[0.479-0.739]	0.862	[0.771-0.917]
Pears	0.577	0.084	< 0.001	[0.413-0.740]	0.841	[0.737-0.904]
Bananas	0.600	0.064	< 0.001	[0.474-0.726]	0.886	[0.783-0.937]
Dried fruit	0.437	0.084	< 0.001	[0.273-0.601]	0.701	[0.502-0.820]
Peaches, nectarines, or plums	0.646	0.078	< 0.001	[0.493-0.798]	0.851	[0.752-0.910]
Grapes	0.514	0.075	< 0.001	[0.367-0.660]	0.755	[0.595-0.852]
Cantaloupe, mango, or papaya	0.453	0.078	< 0.001	[0.299-0.607]	0.692	[0.480-0.816]
Watermelon or melon, other than cantaloupe	0.548	0.064	< 0.001	[0.421-0.674]	0.840	[0.735-0.903]
Strawberries	0.491	0.081	< 0.001	[0.331-0.651]	0.671	[0.457-0.801]
Oranges, tangerines, or clementines	0.464	0.071	< 0.001	[0.325-0.604]	0.803	[0.667-0.883]
Pineapple	0.558	0.089	< 0.001	[0.384-0.733]	0.759	[0.601-0.855]
Other kinds of fruit	0.496	0.078	< 0.001	[0.342-0.650]	0.725	[0.542-0.835]
Median	0.548				0.803	

[a] Weighted Kappa. [b] Standard Error. [c] P-value. [d] Confidence Interval. [e] Intraclass Correlation Coefficient

Table S4. Test–retest reliability of the intake portion of the fruits group

Fruits	Kw [a]	SE[b]	*p*[c]	95% CI[d]	ICC[e]	95% CI[d]
Rutab	0.609	0.190	0.002	[0.238-0.981]	0.820	[0.347-0.877]
Apples	0.541	0.098	< 0.001	[0.349-0.734]	0.783	[0.614-0.878]
Pears	0.641	0.125	< 0.001	[0.396-0.887]	0.845	[0.623-0.936]
Bananas	0.369	0.117	0.002	[0.139-0.599]	0.608	[0.333-0.770]
Dried fruit	0.545	0.130	< 0.001	[0.290-0.801]	0.800	[0.577-0.905]
Peaches, nectarines, or plums	0.349	0.226	0.072	[-0.094-0.792]	0.516	[-0.357-0.830]
Grapes	0.429	0.129	0.003	[0.176-0.681]	0.606	[0.277-0.786]
Cantaloupe, mango, or papaya	0.466	0.120	0.001	[0.231-0.701]	0.730	[0.465-0.865]
Watermelon or melon, other than cantaloupe	0.122	0.112	0.321	[-0.097-0.342]	0.325	[-0.313-0.651]
Strawberries	0.238	0.143	0.105	[-0.043-0.519]	0.363	[-0.239-0.671]
Oranges, tangerines, or clementines	0.362	0.101	0.000	[0.164-0.560]	0.671	[0.436-0.808]
Pineapple	0.520	0.209	0.008	[0.111-0.929]	0.784	[0.223-0.938]
Other kinds of fruit	0.405	0.130	0.001	[0.150-0.659]	0.696	[0.396-0.849]
Median	0.429				0.696	

[a] Weighted Kappa. [b] Standard Error. [c] P-value. [d] Confidence Interval. [e] Intraclass Correlation Coefficient

Table S5: Test–retest reliability of intake frequency of the vegetables, potatoes, and dried beans group

Vegetables, potatoes, dried beans	Kw [a]	SE[b]	*p*[c]	95% CI[d]	ICC[e]	95% CI[d]
Cooked greens	0.305	0.090	< 0.001	[0.129-0.482]	0.555	[0.271-0.730]
Raw greens	0.499	0.071	< 0.001	[0.359-0.638]	0.784	[0.570-0.883]
Coleslaw	0.419	0.153	< 0.001	[0.120-0.719]	0.493	[0.164-0.693]
Cabbage or zucchini	0.506	0.097	< 0.001	[0.315-0.696]	0.802	[0.671-0.881]
Carrots	0.561	0.073	< 0.001	[0.418-0.703]	0.862	[0.772-0.917]
String beans, green beans, or okra	0.290	0.098	< 0.001	[0.098-0.482]	0.301	[-0.169-0.581]
Peas	0.536	0.112	< 0.001	[0.316-0.756]	0.649	[0.416-0.789]
Corn	0.533	0.085	< 0.001	[0.368-0.699]	0.818	[0.698-0.890]
Mushroom	0.494	0.109	< 0.001	[0.280-0.708]	0.584	[0.307-0.750]
Broccoli	0.443	0.154	< 0.001	[0.141-0.746]	0.813	[0.686-0.888]
Cauliflower	0.527	0.080	< 0.001	[0.370-0.683]	0.795	[0.659-0.877]
Winter squash	0.639	0.083	< 0.001	[0.476-0.802]	0.864	[0.774-0.918]
Mixed vegetables	0.423	0.075	< 0.001	[0.277-0.570]	0.687	[0.478-0.812]
Onions	0.593	0.062	< 0.001	[0.473-0.714]	0.866	[0.749-0.925]
Fats added during cooking	0.454	0.081	< 0.001	[0.296-0.612]	0.730	[0.545-0.839]
Fats added after cooking	0.190	0.082	0.023	[0.030-0.350]	0.310	[-0.131]
Sweet peppers	0.676	0.066	< 0.001	[0.546-0.805]	0.873	[0.783-0.925]
Fresh tomatoes	0.580	0.065	< 0.001	[0.452-0.708]	0.858	[0.751-0.917]
Lettuce salads	0.426	0.081	< 0.001	[0.268-0.584]	0.670	[0.449-0.802]
Salad dressing	0.481	0.127	< 0.001	[0.232-0.730]	0.701	[0.506-0.820]
Sweet potatoes	0.469	0.081	< 0.001	[0.310-0.629]	0.621	[0.376-0.770]
French fries	0.397	0.069	< 0.001	[0.262-0.531]	0.684	[0.431-0.819]
Potatoes	0.448	0.071	< 0.001	[0.310-0.587]	0.759	[0.600-0.855]
Tomato sauce or chutney	0.579	0.069	< 0.001	[0.445-0.714]	0.822	[0.630-0.906]
Ketchup	0.555	0.063	< 0.001	[0.432-0.678]	0.814	[0.659-0.894]
Stuffed vegetables	0.606	0.084	< 0.001	[0.441-0.772]	0.810	[0.685-0.886]
Cooked dried beans	0.366	0.081	< 0.001	[0.208-0.524]	0.691	[0.422-0.827]
Other kinds of vegetables	0.365	0.100	< 0.001	[0.169-0.562]	0.536	[0.240-0.718]
Median	0.488				0.745	

[a] Weighted Kappa. [b] Standard Error. [c] P-value. [d] Confidence Interval. [e] Intraclass Correlation Coefficient

Table S6: Test–retest reliability of the intake portion of the vegetables, potatoes, and dried beans group

Vegetables, potatoes, dried beans	Kw [a]	SE[b]	*p*[c]	95% CI[d]	ICC[e]	95% CI[d]
Cooked greens	0.034	0.214	0.872	[-0.385-0.454]	-0.140	[-2.245-0.622]
Raw greens	0.287	0.117	0.015	[0.057-0.516]	0.565	[0.169-0.773]
Coleslaw	0.545	0.362	0.171	[-0.164-1.255]	0.750	[-0.877-0.973]
Cabbage or zucchini	0.472	0.137	0.002	[0.203-0.740]	0.742	[0.383-0.894]
Carrots	0.317	0.134	0.016	[0.055-0.580]	0.470	[-0.044-0.736]
String beans, green beans, or okra	0.371	0.182	0.032	[0.013-0.720]	0.665	[0.009-0.884]
Peas	-	0.242	0.898	[-0.506-0.443]	0.118	[-3.314--.777]
Corn	0.414	0.124	< 0.001	[0.171-0.656]	0.648	[0.346-0.810]
Mushroom	0.340	0.230	0.124	[-0.111-0.790]	0.381	[-0.830-0.798]
Broccoli	-	-	-	-	-	-
Cauliflower	0.432	0.199	0.023	[0.043-0.822]	0.653	[0.046-0.872]
Winter squash	0.474	0.216	0.037	[0.050-0.897]	0.769	[-0.007-0.944]
Mixed vegetables	0.510	0.126	< 0.001	[0.263-0.756]	0.574	[0.129-0.794]
Onions	0.506	0.107	< 0.001	[0.297-0.715]	0.720	[0.461-0.853]
Fats added during cooking	0.401	0.124	0.002	[0.158-0.644]	0.672	[0.372-0.829]
Fats added after cooking	0.508	0.118	< 0.001	[0.278-0.739]	0.732	[0.490-0.859]
Sweet peppers	0.364	0.159	0.025	[0.053-0.675]	0.610	[0.254-0.796]
Fresh tomatoes	0.379	0.112	0.001	[0.160-0.597]	0.711	[0.460-0.846]
Lettuce salads	0.443	0.133	< 0.001	[0.182-0.703]	0.630	[0.0.249-0.820]
Salad dressing	0.450	0.194	0.026	[0.069-0.831]	0.605	[-0.220-0.888]
Sweet potatoes	0.469	0.081	< 0.001	[0.310-0.629]	0.653	[0.126-0.862]
French fries	0.404	0.122	0.002	[0.165-0.644]	0.481	[0.098-0.701]
Potatoes	0.493	0.132	0.001	[0.234-0.752]	0.622	[0.182-0.824]
Tomato sauce or chutney	0.430	0.131	< 0.001	[0.174-0.686]	0.688	[0.421-0.831]
Ketchup	0.323	0.152	0.019	[0.025-0.620]	0.564	[0.174-0.771]
Stuffed vegetables	0.430	0.136	< 0.001	[0.164-0.697]	0.713	[0.250-0.888]
Cooked dried beans	0.321	0.111	0.002	[0.103-0.539]	0.579	[0.249-0.765]
Other kinds of vegetables	0.304	0.156	0.046	[-0.002-0.609]	0.613	[0.130-0.827]
Median	0.422				0.639	

[a] Weighted Kappa. [b] Standard Error. [c] P-value. [d] Confidence Interval. [e] Intraclass Correlation Coefficient

Table S7: Test–retest reliability of the intake frequency of the soups and Middle Eastern foods group

Soups and Middle Eastern foods	Kw [a]	SE[b]	*p*[c]	95% CI[d]	ICC[e]	95% CI[d]
Keema	0.491	0.086	< 0.001	[0.322-0.660]	0.745	[0.578-0.846]
Shawarma	0.601	0.063	< 0.001	[0.478-0.724]	0.807	[0.675-0.885]
Soups	0.652	0.060	< 0.001	[0.535-0.769]	0.907	[0.846-0.944]
Median	0.601				0.807	

[a] Weighted Kappa. [b] Standard Error. [c] P-value. [d] Confidence Interval. [e] Intraclass Correlation Coefficient

Table S8: Test–retest reliability of the intake portion of the soups and middle eastern foods group

Soups and Middle Eastern foods	Kw [a]	SE[b]	*p*[c]	95% CI[d]	ICC[e]	95% CI[d]
Keema	0.292	0.237	0.098	[-0.173-0.756]	-0.026	[-2.193-0.643]
Shawarma	0.580	0.136	< 0.001	[0.314-0.846]	0.763	[0.588-0.864]
Soups	0.747	0.119	< 0.001	[0.513-0.982]	0.878	[0.764-0.937]
Median	0.580				0.821	

[a] Weighted Kappa. [b] Standard Error. [c] P-value. [d] Confidence Interval. [e] Intraclass Correlation Coefficient

Table S9: Test–retest reliability of the intake frequency of the rice, pasta, and pizza group

Rice, pasta, pizza	Kw [a]	SE[b]	*p*[c]	95% CI[d]	ICC[e]	95% CI[d]
White rice	0.345	0.082	< 0.001	[0.184-0.506]	0.607	[0.355-0.762]
Machboos, qabooli, or mandi rice	0.416	0.076	< 0.001	[0.267-0.564]	0.689	[0.480-0.814]
Biryani rice	0.445	0.069	< 0.001	[0.309-0.580]	0.720	[0.535-0.832]
Ersiya or madrouba	0.496	0.075	< 0.001	[0.349-0.644]	0.747	[0.581-0.847]
Pasta	0.519	0.072	< 0.001	[0.378-0.660]	0.729	[0.550-0.837]
Indomie or noodles	0.719	0.062	< 0.001	[0.597-0.840]	0.902	[0.837-0.941]
Koshary	0.542	0.152	< 0.001	[0.245-0.839]	0.473	[0.121-0.684]
Macaroni salad	0.030	0.076	0.731	[-0.118-0.179]	-0.127	[-0.888-0.325]
Pizza	0.476	0.086	< 0.001	[0.308-0.644]	0.649	[0.416-0.789]
Median	0.476				0.705	

[a] Weighted Kappa. [b] Standard Error. [c] P-value. [d] Confidence Interval. [e] Intraclass Correlation Coefficient

Table S10: Test–retest reliability of the intake portion of the rice, pasta, and pizza group

Rice, pasta, pizza	Kw [a]	SE[b]	*p*[c]	95% CI[d]	ICC[e]	95% CI[d]
White rice	0.362	0.121	0.001	[0.125-0.598]	0.574	[0.249-0.759]
Machboos, qabooli, or mandi rice	0.429	0.103	< 0.001	[0.226-0.631]	0.597	[0.329-0.758]
Biryani rice	0.434	0.121	< 0.001	[0.197-0.671]	0.522	[0.157-0.732]
Ersiya or madrouba	0.496	0.137	< 0.001	[0.227-0.765]	0.708	[0.359-0.868]
Pasta	0.372	0.122	< 0.001	[0.133-0.611]	0.637	[0.338-0.802]
Indomie or noodles	0.426	0.165	0.001	[0.101-0.750]	0.674	[0.321-0.846]
Koshary	0.609	0.240	0.006	[0.139-1.078]	0.818	[0.126-0.960]
Macaroni salad	-	0.306	0.386	[-1.100-0.100]	-	-
Pizza	0.531	0.135	< 0.001	[0.267-0.796]	0.739	[0.505-0.863]
Median	0.432				0.656	

[a] Weighted Kappa. [b] Standard Error. [c] P-value. [d] Confidence Interval. [e] Intraclass Correlation Coefficient

Table S11: Test–retest reliability of the intake frequency of the cereal, qaroos, and breads group

Cereal, qaroos, breads	Kw [a]	SE[b]	*p*[c]	95% CI[d]	ICC[e]	95% CI[d]
Harees or jareesh	0.582	0.077	< 0.001	[0.430-0.734]	0.789	[0.649-0.873]
Oatmeal or other cooked cereal	0.579	0.066	< 0.001	[0.450-0.709]	0.847	[0.746-0.907]
Breakfast cereal	0.419	0.135	< 0.001	[0.155-0.682]	0.726	[0.545-0.835]
Qaroos, pancake, french toast or waffles	0.393	0.077	< 0.001	[0.241-0.544]	0.692	[0.461-0.820]
Paratha, chapati, or marduf bread	0.355	0.076	< 0.001	[0.205-0.505]	0.613	[0.353-0.768]
Bread as part of a sandwich	0.225	0.077	0.002	[0.074-0.375]	0.482	[0.158-0.684]
Bread NOT as part of a sandwich	0.431	0.079	< 0.001	[0.277-0.586]	0.635	[0.398-0.779]
Thareed	0.633	0.119	< 0.001	[0.400-0.865]	0.796	[0.661-0.877]
Median	0.425				0.709	

[a] Weighted Kappa. [b] Standard Error. [c] P-value. [d] Confidence Interval. [e] Intraclass Correlation Coefficient

Table S12: Test–retest reliability of the intake portion of the cereal, qaroos, and breads group

**Cereal, qaroos, breads**	**Kw** [a]	**SE**[b]	***p***[c]	**95% CI**[d]	**ICC**[e]	**95% CI**[d]
Harees or jareesh	0.401	0.153	0.007	[0.102-0.700]	0.639	[0.167-0.842]
Oatmeal or other cooked cereal	0.385	0.167	0.008	[0.057-0.712]	0.537	[-0.126-0.814]
Breakfast cereal	0.400	0.283	0.157	[-0.154-0.954]	0.593	[-0.482-0.884]
Qaroos, pancake, french toast or waffles	0.524	0.101	< 0.001	[0.326-0.722]	0.731	[0.516-0.850]
Paratha, chapati, or marduf bread	0.614	0.118	< 0.001	[0.382-0.846]	0.773	[0.602-0.870]
Bread as part of a sandwich	0.468	0.095	< 0.001	[0.282-0.654]	0.739	[0.534-0.853]
Bread NOT as part of a sandwich	0.326	0.137	0.013	[0.058-0.594]	0.606	[0.606-0.809]
Thareed	0.231	0.290	0.270	[-0.337-0.799]	-	-
Median	0.401				0.639	

[a] Weighted Kappa. [b] Standard Error. [c] P-value. [d] Confidence Interval. [e] Intraclass Correlation Coefficient

Table S13: Test–retest reliability of the intake frequency of the date molasses, jam, and peanut butter group

**Date molasses, jam, peanut butter**	**Kw** [a]	**SE**[b]	***p***[c]	**95% CI**[d]	**ICC**[e]	**95% CI**[d]
Date molasses, honey or jam	0.491	0.078	< 0.001	[0.338-0.643]	0.706	[0.512-0.822]
Peanut butter or other nut butter	0.534	0.110	< 0.001	[0.318-0.750]	0.763	[0.607-0.857]
Median	0.513				0.735	

[a] Weighted Kappa. [b] Standard Error. [c] P-value. [d] Confidence Interval. [e] Intraclass Correlation Coefficient

Table S14: Test–retest reliability of the intake portion of the date molasses, jam, and peanut butter group

**Date molasses, jam, peanut butter**	**Kw** [a]	**SE**[b]	***p***[c]	**95% CI**[d]	**ICC**[e]	**95% CI**[d]
Date molasses, honey or jam	0.502	0.191	< 0.001	[0.127-0.877]	0.718	[0.409-0.866]
Peanut butter or other nut butter	0.421	0.190	0.029	[0.048-0.794]	0.737	[0.114-0.927]
Median	0.462				0.728	

[a] Weighted Kappa. [b] Standard Error. [c] P-value. [d] Confidence Interval. [e] Intraclass Correlation Coefficient

Table S15: Test–retest reliability of the intake frequency of the mashakik, shiwa, and cold cuts group

**Mashakik, shiwa, cold cuts**	**Kw** [a]	**SE**[b]	***p***[c]	**95% CI**[d]	**ICC**[e]	**95% CI**[d]
Mishkak or shiwa meat in sandwiches	0.426	0.092	< 0.001	[0.246-0.607]	0.627	[0.380-0.776]
Chicken or Turkey cold cuts	0.331	0.086	< 0.001	[0.163-0.500]	0.525	[0.224-0.711]
Other cold cuts or luncheon meats	0.292	0.094	0.001	[0.108-0.477]	0.419	[0.056-0.645]
Hot dog or frankfurters	0.603	0.069	< 0.001	[0.468-0.738]	0.829	[0.717-0.897]
Median	0.379				0.576	

[a] Weighted Kappa. [b] Standard Error. [c] P-value. [d] Confidence Interval. [e] Intraclass Correlation Coefficient

Table S16: Test–retest reliability of the intake portion of the mashakik, shiwa, and cold cuts group

**Mashakik, shiwa, cold cuts**	**Kw** [a]	**SE**[b]	***p***[c]	**95% CI**[d]	**ICC**[e]	**95% CI**[d]
Mishkak or shiwa meat in sandwiches	0.483	0.101	< 0.001	[0.284-0.682]	0.713	[0.417-0.853]
Chicken or Turkey cold cuts	0.723	0.130	< 0.001	[0.467-0.979]	0.870	[0.718-0.940]
Other cold cuts or luncheon meats	0.107	0.254	0.647	[-0.390-0.604]	-0.145	[-5.911-0.737]
Hot dog or frankfurters	0.323	0.194	0.063	[-0.058-0.703]	0.528	[-0.053-0.787]
Median	0.403				0.713	

[a] Weighted Kappa. [b] Standard Error. [c] P-value. [d] Confidence Interval. [e] Intraclass Correlation Coefficient

Table S17: Test–retest reliability of the intake frequency of the meat, poultry, and fish group

Meat, poultry, fish	Kw [a]	SE[b]	*p*[c]	95% CI[d]	ICC[e]	95% CI[d]
Chicken burger, chicken kebab, or minced	0.525	0.076	< 0.001	[0.376-0.675]	0.758	[0.600-0.854]
Beef burgers from a fast food or other restaurant	0.432	0.090	< 0.001	[0.256-0.608]	0.565	[0.276-0.739]
Beef burgers NOT from a fast food or other restaurant	0.492	0.088	< 0.001	[0.319-0.665]	0.752	[0.588-0.851]
Ground beef in mixtures	0.467	0.080	< 0.001	[0.310-0.624]	0.743	[0.574-0.845]
Beef mixtures	0.392	0.084	< 0.001	[0.227-0.557]	0.636	[0.396-0.781]
Mishkak or shiwa meat	0.483	0.079	< 0.001	[0.329-0.637]	0.790	[0.639-0.876]
Steak	0.501	0.123	< 0.001	[0.260-0.741]	0.716	[0.529-0.828]
Ribs (meat)	0.572	0.103	< 0.001	[0.371-0.773]	0.713	[0.524-0.827]
Chicken mixtures	0.343	0.075	< 0.001	[0.196-0.490]	0.657	[0.430-0.793]
Baked, broiled, roasted, stewed, or fried chicken	0.322	0.087	< 0.001	[0.152-0.492]	0.416	[0.034-0.647]
Quail	0.359	0.210	< 0.001	[-0.053-0.770]	0.450	[0.086-0.669]
Liver	0.670	0.070	< 0.001	[0.533-0.807]	0.861	[0.769-0.916]
Qaliya or mueajin	0.518	0.095	< 0.001	[0.332-0.703]	0.657	[0.429-0.793]
Sausage	0.024	0.075	< 0.001	[-0.122-0.170]	- 0.079	[-0.812-0.354]
Canned tuna	0.678	0.061	< 0.001	[0.557-0.798]	0.876	[0.794-0.925]
Fried shellfish (such as shrimp, lobster, or crab)	0.469	0.102	< 0.001	[0.268-0.670]	0.651	[0.418-0.790]
Shellfish (such as shrimp, lobster, or crab) that was NOT fried	0.709	0.089	< 0.001	[0.534-0.884]	0.832	[0.722-0.899]
Fish, such as (sahwa/ gaydher (tuna), kanaad, sal, dhala/karufa, sardine, or salmon)	0.538	0.070	< 0.001	[0.401-0.674]	0.818	[0.697-0.890]
Dried fish, such as (cured fish (malih), dried anchovies (kasha), or dried shark (awal)	0.423	0.103	< 0.001	[0.220-0.626]	0.675	[0.459-0.804]
Fish sticks or other fried fish (not including shellfish)	0.287	0.126	0.004	[0.040-0.534]	0.569	[0.282-0.741]
Other fish that was NOT fried (not including shellfish)	0.215	0.105	0.017	[0.010-0.420]	0.357	[-0.041-0.607]
Fats used to fry, sauté, baste, or marinate any meat, poultry, or fish	0.379	0.085	< 0.001	[0.212-0.546]	0.689	[0.485-0.813]
Median	0.468				0.689	

[a] Weighted Kappa. [b] Standard Error. [c] P-value. [d] Confidence Interval. [e] Intraclass Correlation Coefficient

Table S18: Test–retest reliability of the intake portion of the meat, poultry, and fish group

**Meat, poultry, fish**	**Kw** [a]	**SE**[b]	***p***[c]	**95% CI**[d]	**ICC**[e]	**95% CI**[d]
Chicken burger, chicken kebab, or minced	0.377	0.129	0.001	[0.125-0.630]	0.658	[0.364-0.816]
Beef burgers from a fast food or other restaurant	0.544	0.145	< 0.001	[0.261-0.828]	0.727	[0.454-0.863]
Beef burgers NOT from a fast food or other restaurant	0.397	0.184	0.049	[0.036-0.758]	0.579	[0.028-0.820]
Ground beef in mixtures	0.248	0.150	0.059	[-0.047-0.542]	0.508	[-0.046-0.767]
Beef mixtures	0.588	0.110	< 0.001	[0.373-0.803]	0.792	[0.634-0.882]
Mishkak or shiwa meat	0.481	0.116	< 0.001	[0.253-0.709]	0.739	[0.515-0.860]
Steak	0.696	0.268	0.053	[0.170-1.222]	0.842	[0.189-0.972]
Ribs (meat)	0.844	0.156	0.001	[0.538-1.150]	0.947	[0.799-0.987]
Chicken mixtures	0.258	0.110	0.007	[0.042-0.474]	0.512	[0.175-0.711]
Baked, broiled, roasted, stewed, or fried chicken	0.596	0.106	< 0.001	[0.388-0.805]	0.781	[0.588-0.883]
Quail	-	-	-	-	-	-
Liver	0.505	0.157	0.004	[0.197-0.813]	0.770	[0.426-0.907]
Qaliya or mueajin	0.458	0.165	0.004	[0.134-0.781]	0.708	[0.270-0.882]
Sausage	-	-	-	-	-	-
Canned tuna	0.304	0.165	0.042	[-0.019-0.627]	0.487	[-0.081-0.755]
Fried shellfish (such as shrimp, lobster, or crab)	0.476	0.209	0.010	[0.067-0.885]	0.737	[0.065-0.928]
Shellfish (such as shrimp, lobster, or crab) that was NOT fried	0.349	0.231	0.068	[-0.103-0.801]	0.665	[0.009-0.884]
Fish, such as (sahwa/ gaydher (tuna), kanaad, sal, dhala/karufa, sardine, or salmon)	0.508	0.103	< 0.001	[0.306-0.711]	0.754	[0.575-0.857]
Dried fish, such as (cured fish (malih), dried anchovies (kasha), or dried shark (awal)	0.517	0.200	0.005	[0.125-0.910]	0.764	[0.287-0.923]
Fish sticks or other fried fish (not including shellfish)	0.500	0.375	0.248	[-0.235-1.235]	0.727	[-1.809-0.982]
Other fish that was NOT fried (not including shellfish)	0.029	0.207	0.887	[-0.376-0.434]	0.167	[-2.310-0.780]
Median	0.481				0.727	

[a] Weighted Kappa. [b] Standard Error. [c] P-value. [d] Confidence Interval. [e] Intraclass Correlation Coefficient

Table S19: Test–retest reliability of the intake frequency of the eggs and meat alternatives group

Eggs and meat alternatives	Kw [a]	SE[b]	*p*[c]	95% CI[d]	ICC[e]	95% CI[d]
Tofu, soy burgers, or soy meat substitutes	-	0.016	0.854	[-0.053-0.009]	-0.046	[-0.749-0.373]
Eggs	0.398	0.088	< 0.001	[0.225-0.571]	0.569	[0.293-0.738]
Median	0.398				0.569	

[a] Weighted Kappa. [b] Standard Error. [c] P-value. [d] Confidence Interval. [e] Intraclass Correlation Coefficient

Table S20: Test–retest reliability of the intake portion of the eggs and meat alternatives group

Eggs and meat alternatives	Kw [a]	SE[b]	*p*[c]	95% CI[d]	ICC[e]	95% CI[d]
Tofu, soy burgers, or soy meat substitutes	-	-	-	-	-	-
Eggs	0.551	0.093	< 0.001	[0.369-0.733]	0.793	[0.642-0.881]
Median	0.551				0.793	

[a] Weighted Kappa. [b] Standard Error. [c] P-value. [d] Confidence Interval. [e] Intraclass Correlation Coefficient

Table S21: Test-retest reliability of the intake frequency of the chips, rusk, and other snacks group

Chips, Rusk, and other snacks	Kw [a]	SE[b]	*p*[c]	95% CI[d]	ICC[e]	95% CI[d]
Crackers	0.587	0.087	< 0.001	[0.418-0.757]	0.832	[0.713-0.901]
Potato chips	0.347	0.080	< 0.001	[0.190-0.504]	0.581	[0.312-0.746]
Tortilla chips	0.278	0.112	< 0.001	[0.057-0.498]	0.524	[0.208-0.714]
Popcorn	0.460	0.091	< 0.001	[0.281-0.639]	0.766	[0.611-0.859]
Rusk (baksam) or tea biscuits, like Nabil and Marie	0.590	0.094	< 0.001	[0.406-0.773]	0.860	[0.767-0.916]
Nuts and seeds	0.569	0.080	< 0.001	[0.413-0.725]	0.868	[0.775-0.922]
Energy, high-protein, or breakfast bars	0.524	0.162	< 0.001	[0.206-0.842]	0.773	[0.623-0.864]
Median	0.524				0.773	

[a] Weighted Kappa. [b] Standard Error. [c] P-value. [d] Confidence Interval. [e] Intraclass Correlation Coefficient

Table S22: Test–retest reliability of the intake portion of the chips, rusk, and other snacks group

**Chips, rusk, and other snacks**	**Kw [a]**	**SE[b]**	***p*[c]**	**95% CI[d]**	**ICC[e]**	**95% CI[d]**
Crackers	0.347	0.189	0.044	[-0.023-0.717]	0.624	[0.095-0.842]
Potato chips	0.377	0.137	0.001	[0.108-0.645]	0.542	[0.172-0.746]
Tortilla chips	0.600	0.232	0.038	[0.146-1.054]	0.769	[0.170-0.941]
Popcorn	0.639	0.151	< 0.001	[0.343-0.936]	0.866	[0.670-0.946]
Rusk (baksam) or tea biscuits, like Nabil and Marie	0.204	0.199	0.273	[-0.186-0.593]	0.452	[-0.480-0.792]
Nuts and seeds	0.655	0.098	< 0.001	[0.462-0.847]	0.848	[0.718-0.919]
Energy, high-protein, or breakfast bars	-	-	-	-	-	-
Median	0.489				0.697	

[a] Weighted Kappa. [b] Standard Error. [c] P-value. [d] Confidence Interval. [e] Intraclass Correlation Coefficient

Table S23: Test–retest reliability of the intake frequency of the yoghurt and cheese group

**Yoghurt and cheese**	**Kw [a]**	**SE[b]**	***p*[c]**	**95% CI[d]**	**ICC[e]**	**95% CI[d]**
Yoghurt or laban, not laban up	0.422	0.082	< 0.001	[0.262-0.582]	0.738	[0.567-0.842]
Laban up	0.500	0.082	< 0.001	[0.340-0.660]	0.780	[0.637-0.868]
kami cheese, feta cheese, cottage cheese, white cheese	0.432	0.103	< 0.001	[0.230-0.634]	0.694	[0.491-0.816]
Spreadable cream cheese, triangle cheese, cheese slices	0.315	0.072	< 0.001	[0.173-0.456]	0.617	[0.366-0.769]
Median	0.427				0.716	

[a] Weighted Kappa. [b] Standard Error. [c] P-value. [d] Confidence Interval. [e] Intraclass Correlation Coefficient

Table S24: Test–retest reliability of the intake portion of the yoghurt and cheese group

**Yoghurt and cheese**	**Kw [a]**	**SE[b]**	***p*[c]**	**95% CI[d]**	**ICC[e]**	**95% CI[d]**
Yoghurt or laban, not laban up	0.409	0.125	< 0.001	[0.164-0.656]	0.675	[0.426-0.817]
Laban up	0.378	0.172	0.016	[0.041-0.715]	0.554	[-0.007-0.801]
kami cheese, feta cheese, cottage cheese, white cheese	-	0.194	0.675	[-0.467-0.293]	0.146	[-1.731-0.720]
Spreadable cream cheese, triangle cheese, cheese slices	0.141	0.155	0.255	[-0.162-0.444]	0.236	[-0.467-0.601]
Median	0.259				0.395	

[a] Weighted Kappa. [b] Standard Error. [c] P-value. [d] Confidence Interval. [e] Intraclass Correlation Coefficient

Table S25: Test–retest reliability of the intake frequency of the sweets, baked goods, and desserts group

Sweets, baked goods, desserts	Kw[a]	SE[b]	p[c]	95% CI[d]	ICC[e]	95% CI[d]
Addition of sugar or honey to foods	0.311	0.087	< 0.001	[0.141-0.480]	0.523	[0.211-0.712]
Frozen yogurt	0.360	0.142	0.001	[0.082-0.637]	0.578	[0.296-0.746]
Ice cream	0.485	0.080	< 0.001	[0.328-0.641]	0.791	[0.652-0.874]
Cake or muffin	0.388	0.082	< 0.001	[0.228-0.549]	0.689	[0.473-0.815]
Cookies or brownies	0.402	0.078	< 0.001	[0.249-0.555]	0.744	[0.576-0.845]
Doughnuts, cinnamon rolls, or knafeh	0.502	0.071	< 0.001	[0.364-0.640]	0.767	[0.615-0.859]
Luqaimat or balah al sham	0.339	0.093	0.001	[0.157-0.521]	0.614	[0.357-0.768]
Rahash or baklava	0.431	0.093	< 0.001	[0.249-0.612]	0.579	[0.305-0.745]
Croissants, lolaa, mandazi, maamoul, asida, khabees	0.366	0.073	< 0.001	[0.224-0.508]	0.702	[0.505-0.821]
Fruit cake	0.329	0.094	< 0.001	[0.145-0.513]	0.601	[0.335-0.760]
Pistachio or other nut cake	0.119	0.123	0.272	[-0.123-0.361]	0.180	[-0.371-0.508]
Chocolate	0.473	0.074	< 0.001	[0.327-0.619]	0.773	[0.625-0.863]
Candy	0.508	0.123	< 0.001	[0.268-0.749]	0.834	[0.726-0.900]
Omani halwa	0.662	0.072	< 0.001	[0.522-0.802]	0.833	[0.720-0.900]
Median	0.395				0.695	

[a] Weighted Kappa. [b] Standard Error. [c] P-value. [d] Confidence Interval. [e] Intraclass Correlation Coefficient

Table S26: Test–retest reliability of the intake portion of the sweets, baked goods, and desserts group

Sweets, baked goods, desserts	Kw[a]	SE[b]	p[c]	95% CI[d]	ICC[e]	95% CI[d]
Addition of sugar or honey to foods	-	0.125	0.685	[-0.303-0.185]	-0.048	[-1.641-0.568]
Frozen yogurt	0.706	0.204	0.025	[0.306-1.106]	0.889	[0.176-0.988]
Ice cream	0.151	0.160	0.297	[-0.164-0.465]	0.375	[-0.391-0.717]
Cake or muffin	0.402	0.161	0.002	[0.087-0.718]	0.582	[0.115-0.802]
Cookies or brownies	0.220	0.173	0.193	[-0.118-0.559]	0.493	[-0.256-0.792]
Doughnuts, cinnamon rolls, or knafeh	0.596	0.115	< 0.001	[0.370-0.821]	0.817	[0.624-0.911]
Luqaimat or balah al sham	0.224	0.194	0.136	[-0.155-0.604]	0.328	[-0.659-0.736]
Rahash or baklava	0.354	0.181	0.079	[0.000-0.708]	0.625	[0.041-0.860]
Croissants, lolaa, mandazi, maamoul, asida, khabees	0.197	0.123	0.071	[-0.045-0.439]	0.464	[0.022-0.706]
Fruit cake	0.189	0.202	0.270	[-0.206-0.584]	0.300	[-1.314-0.773]
Pistachio or other nut cake	1.000	0.000	0.157	[1.000-1.000]	1.000	-
Chocolate	0.469	0.123	0.001	[0.227-0.711]	0.660	[0.377-0.815]
Candy	0.842	0.155	0.001	[0.538-1.146]	0.943	[0.766-0.987]
Omani halwa	0.438	0.145	< 0.001	[0.153-0.722]	0.621	[0.267-0.805]
Median	0.402				0.621	

[a] Weighted Kappa. [b] Standard Error. [c] P-value. [d] Confidence Interval. [e] Intraclass Correlation Coefficient
